# The neutrophil-lymphocyte ratio and its relationship with insulin resistance in obesity

**DOI:** 10.3906/sag-1804-68

**Published:** 2019-02-11

**Authors:** Serdar KARAKAYA, Mustafa ALTAY, Fatma KAPLAN EFE, İbrahim KARADAĞ, Oktay ÜNSAL, Oktay BULUR, Murat ESER, Derun TANER ERTUĞRUL

**Affiliations:** 1 Department of Medical Oncology, University of Health Sciences,Dr. A. Yurtaslan Health Administration and Research Center, Ankara Turkey; 2 Department of Endocrinology and Metabolism, University of Health Sciences,Keçiören Health Administration and Research Center, Ankara Turkey; 3 Department of Internal Medicine, University of Health Sciences,Keçiören Health Administration and Research Center, Ankara Turkey

**Keywords:** Neutrophil-lymphocyte ratio, obesity, insulin resistance

## Abstract

**Background/aim:**

In this study, our aim was to investigate the neutrophil/lymphocyte (N/L) ratio, variations in leukocytes and leukocyte subtypes, and the relationship between N/L ratio and insulin resistance (IR) in obesity.

**Materials and methods:**

Ninety-six patients and 40 healthy controls were included in this study. Patients’ blood glucose levels, insulin levels, and hemogram parameters upon 8 h of fasting were determined. Body mass index (BMI) and Homeostasis Model Assessment-Insulin Resistance (HOMA-IR) values were calculated.

**Results:**

Neutrophil numbers were found to be higher among obese patients with IR than among non-IR obese patients. The N/L ratio was, moreover, found to be higher among obese patients with IR when compared to non-IR obese patients. A positive correlation was found between insulin resistance and both neutrophil and WBC counts. Positive correlations were also found between insulin levels and the N/L ratio, WBC counts, and neutrophil counts.

**Conclusion:**

In our study, leukocyte numbers and subtypes were determined to be higher among obese individuals than among healthy individuals. The N/L ratio was increased significantly only among obese patients with IR. Further studies are needed in order to better demonstrate the relationship between the N/L ratio and IR/inflammation.

## 1. Introduction

Obesity is increasingly becoming a major public health problem worldwide. Its prevalence has spiked dramatically over the past 20 years. In the United States, 35.7% of adults are obese, while in Turkey 32% of adults are obese (1). Obesity has a negative impact on many tissues and systems in the body that are associated with many various mechanisms. Inflammation is certainly one of the most important of these mechanisms. Obesity-induced microinflammation has been shown in different studies using different biomarkers (2). For example, obesity has been shown to be positively associated with increased levels of CRP, an increased white blood cell (WBC) count, and an increased number of leukocyte subtypes (2). The neutrophil-lymphocyte ratio (N/L ratio) is known to be associated with inflammation. For example, it has been shown to be associated with subclinical inflammation both in certain malignancy types and in coronary artery disease (3–7). Although the role of inflammation in obesity and insulin resistance (IR) is well known, there nevertheless remains a lack of sufficient data with regards to the N/L ratio. Thus, this study has been geared towards investigating how the N/L ratio, leukocytes, and their subtypes are affected among obese patients.

## 2. Materials and methods 

The data from 250 obese patients were scanned retrospectively, out of which 96 patients who fit the inclusion criteria and had complete file information were included in the study. The inclusion criteria were predefined as patients being 18 years of age and older, with a body mass index (BMI) of ≥25 kg/m2; having no systemic diseases such as arterial hypertension, diabetes, or metabolic abnormality; having no active or chronic infectious or inflammatory diseases; and not being a part of any ongoing drug treatment. The inclusion criteria for the control group were defined as being healthy individuals having a BMI of <25 kg/m2, having neither active nor chronic diseases, and not taking part in any ongoing drug treatment. Forty healthy individuals from among 120 people who were admitted to the hospital for check-ups were selected as the control group in accordance with the inclusion criteria. Patients’ demographic data such as age, sex, height, and weight were also recorded. BMI was calculated as follows: BMI = weight (kg)/height squared (m2). Patients’ blood glucose and insulin levels, as well WBC, neutrophil and lymphocyte counts and N/L ratios following a minimum of 8 h of fasting were obtained from their medical records.

Patients’ levels of IR were calculated according to the following formula: Homeostasis Model Assessment (HOMA)-IR= fasting plasma glucose × fasting insulin/405. HOMA-IR of ≥3 is accepted as insulin resistance (8). The results of both the patient and control groups were then recorded into an SPSS database, whereupon all statistical analysis was performed using SPSS 18.0. Parametric tests were used to compare the data between groups and variables. Pearson correlation analysis was used in order to evaluate the relationships between each variable. P < 0.05 was considered to be statistically significant.

## 3. Results

Ninety-six patients and 40 healthy controls were included in this study. There was no statistical difference between the groups in terms of age and sex (Table 1). White blood cell counts were significantly higher in obese patients (8764 ± 2023 and 7712 ± 1932, respectively, P = 0.006). Neutrophil and lymphocyte numbers were also significantly higher among the obese group relative to the control group (neutrophils: 5359 ± 1788 and 4585 ± 1473, respectively, P = 0.02; lymphocytes: 2615 ± 627 and 2287 ± 553, respectively, P = 0.005). However, there was no statistically significant difference for the N/L ratio between groups (2.18 ± 1.00 and 2.10 ± 0.83, respectively, P = 0.7).

**Table 1:** Laboratory parameters of obese and nonobese individuals.

	Control (n = 40)	Obesity (n = 96)	P-value
Age (years)	33.38 ± 9.41	36.75 ± 11.11	0.09
Sex (F/M)	31/9	78/18	0.4
BMI (kg/m2)	22.14 ± 2.61	32.71 ± 5.57	<0.001
Neutrophils (/µL)	4585 ± 1473	5359 ± 1788	0.02
Lymphocytes (/µL)	2287 ± 553	2615 ± 627	0.005
N/L ratio	2.10 ± 0.83	2.18 ± 1.01	0.7
WBCs (/µL)	7712 ± 1932	8764 ± 2023	0.006

BMI: Body mass index, N/L ratio: neutrophil/lymphocyte ratio, WBC: white blood cell.

When the patient group was examined for insulin resistance (HOMA-IR ≥3), there was no statistically significant difference among obese individuals without IR (51 patients in total) and obese individuals with IR (46 patients in total) with respect to age and sex (Table 2). Neutrophil numbers were found to be higher among obese patients with IR than among non-IR obese patients (5780 ± 1628 and 4980 ± 1838, P = 0.02). No statistical difference was found between IR obese and non-IR obese patients in terms of lymphocyte numbers (2595 ± 625 and 2649 ± 637, P = 0.7). The N/L ratio, however, was found to be higher in IR obese patients when compared to their non-IR obese counterparts (2.39 ± 1.06 vs. 1.97 ± 0.91, P = 0.04).

**Table 2:** Laboratory parameters of obese individuals with insulin resistance and obese individuals without insulin resistance.

	Non-IR obese patients(n = 51)	IR obese patients(n = 46)	P-value
Age (years)	36.63 ± 11.20	36.89 ± 11.01	0.9
Sex (F/M)	43/8	36/10	0.08
BMI (kg/m2)	30.52 ± 3.82	35.00 ± 6.37	<0.001
Insulin (mIU/mL)	9.38 ± 2.79	20.32 ± 6.06	<0.001
HOMA-IR	2.04 ± 0.62	4.61 ± 1.44	<0.001
Neutrophils (/µL)	4980 ± 1838	5780 ± 1628	0.03
Lymphocytes (/µL)	2649 ± 637	2595 ± 625	0.9
N/L ratio	1.98 ± 0.91	2.39 ± 1.06	0.04
WBCs (/µL)	8386 ± 2156	9276 ± 1857	0.03

BMI: Body mass index, HOMA-IR: Homeostasis Model Assessment-Insulin Resistance, N/L ratio: neutrophil/lymphocyte ratio, WBC: white blood cell.

Correlation analysis of both the patient and control groups revealed a positive correlation between IR and both neutrophils and WBC counts (neutrophils: r = 0.331, P = 0.001; WBC: r = 0.377, P < 0.001). Other positive correlations were found for N/L ratio, WBC counts, and neutrophil numbers (N/L ratio: r = 0.215, P = 0.03; WBC: r = 0.410, P < 0.001; neutrophils: r = 0.368, P < 0.001).

## 4. Discussion

In our study, we observed that neutrophil and lymphocyte numbers were higher among obese individuals than among nonobese individuals while the N/L ratio was similar between the two groups. However, among obese patients, we observed that as the level of IR increased, so did both the N/L ratio and the number of leukocytes and their subtypes.

The relationship between inflammation and obesity is now very well established. Leukocytes, being responsible for a portion of this relationship, infiltrate fat tissue and release inflammatory cytokines (9,10). Metaanalysis showed that there was an increase in circulating white blood cells among obese patients, thus posing a high risk for type 2 diabetes (11). A relationship was also found between circulating leukocyte subtypes and IR among diabetic people, with additional risk factors (12). Previous studies revealed a strong correlation between leukocyte number (a clinical inflammation marker) and subtypes and obesity (13).

Unlike many previous studies, obese individuals who had no additional risk factor were investigated in our study. We analyzed the relationship between obesity and inflammation in obese and nonobese groups according to both insulin levels and IR. Neutrophil, lymphocyte, and white blood cell numbers were statistically different between the obese and nonobese group. Among the patient group, N/L ratios were found to be significantly higher in those with versus those without IR. This finding shows us that the difference in N/L ratio with obesity is linked to a rise in IR. The lack of statistical significance between the groups, however, may be due to the fact not every obese patient was found have IR.

There was a significant difference in terms of neutrophils and white blood cell counts between the two groups divided according to IR. This shows us that BMI and levels of leukocyte/leukocyte subtypes increase concomitantly.

Therefore, according to our findings, it seems that obesity ought to be considered as one of the causes of an increase in leukocytes and leukocyte subtypes. Similar findings have also been previously documented among diabetic patients. The increase in the number of neutrophils, lymphocytes, and leukocytes in individuals at high risk for developing diabetes has been shown to support subclinical inflammation (14).

The findings of our study furthermore suggest that obese people with no additional risk factors nevertheless have a common mechanism, e.g., diabetes. However, our study reveals that there is an increase in levels of both neutrophils and lymphocytes, which in turn implies that the N/L ratio can explain the further increase in neutrophil number as insulin level increases. What has also been demonstrated is that there is a positive correlation between insulin levels and leukocyte and neutrophil numbers (15). Our study reveals that there is a positive correlation between the N/L ratio and insulin level.

These differences may be due to lack of number of cases in previous studies. These results support that increment in insulin level increases inflammation via inflammatory mediators. The lack of other markers of inflammation (e.g., CRP and sedimentation), coupled by a lack of demographic data (e.g., waist circumference), constitute the limitations of our study.

In conclusion, data emerging from our study indicate that the N/L ratio may be used as a marker for obese individuals who have high IR. Accordingly, the increase in the neutrophil, lymphocyte, and leukocyte numbers among obese patients who lack any additional health complications being a possible result of IR should be kept in mind. However, we believe that further studies are needed in this regard.
